# Supplementary material for: Content-rich biological network constructed by mining PubMed abstracts
Source: BMC Bioinformatics. 2004 Oct 8;5:147. doi: 10.1186/1471-2105-5-147 (PMC528731; doi:10.1186/1471-2105-5-147)
Supplement: Additional File 2 — The original results of the above study (non-essential files are deleted to keep the file size under the limit set by BMC bioinformatics). [file 1471-2105-5-147-S2.bz2 › chilibotAdditionalFile2/dip20/385ID9252374E9850/html/THPO_MPL.html]

 


 **THPO** and **MPL** 
  
Found 539 abstracts in PubMed, retrieved 20.  
 

 What does Google say? 
 PDF only 
| .edu only 

---

**Interactive relationship** (e.g. stimulation, inhibition, etc)

**Stimulatory relationship**- Interestingly, decreased c  **MPL**   [ **MPL** ]  and elevated PRV 1 were also observed in patients with hereditary thrombocythemia HT who carry a mutation in the thrombopoietin  [ **THPO** ]  TPO  [ **THPO** ]  gene.  Ref: 12730106 Blood< MedlineTA>Blood,
- Although it is clear that thrombopoietin  [ **THPO** ]  is the primary regulator of thrombopoiesis, several lines of evidence indicate that the hormone affects multiple aspects of hematopoiesis the in vivo administration of TPO  [ **THPO** ]  increases marrow levels of erythroid, myeloid, and megakaryocytic progenitor cells and its genetic elimination or that of its receptor c  **mpl**   [ **MPL** ]  reduces the numbers of these cells.  Ref: 12799280 Ann N Y Acad Sci< MedlineTA>Ann N Y Acad Sci,
**Neutral relationship**- One mutant  **Mpl**  type SAG Delta GCRMplTmR successfully lost the responsiveness to TPO  [ **THPO** ]  without affecting the Tm dependence.  Ref: 12646182 Biochem Biophys Res Commun, 2003
- The cloning of thrombopoietin  [ **THPO** ]  TPO  [ **THPO** ] , followed by the analysis of TPO  [ **THPO** ]  and c  **mpl**   [ **MPL** ]  its cellular receptor knockout mice confirmed its role as the primary regulator of thrombopoiesis.  Ref: 12650174 J Gravit Physiol, 2001
- Thrombopoietin  [ **THPO** ]  TPO  [ **THPO** ]  is a hematopoietic cytokine that regulates megakaryocytosis and thrombocytosis by binding to its receptor c  **Mpl**   [ **MPL** ] .  Ref: 12841634 Mol Cell Biochem, 2003
- Our case report suggests that AMT in patients with SSc could be mediated by the anti c  **Mpl**   [ **MPL** ]  antibody, which functionally blocks an interaction between thrombopoietin  [ **THPO** ]  and c  **Mpl**   [ **MPL** ] .  Ref: 12794833 Arthritis Rheum, 2003

**Non-interactive relationship** (e.g. studied together, co-existance, homology, etc.)

- Because these proteins were of three different classes rHuEPO is an N linked glycoprotein,  **Mpl**  ligand  [ **THPO** ]  an O linked glycoprotein, and leptin contains no carbohydrate, glycoengineering may be generally applicable as a strategy for increasing the in vivo activity and duration of action of proteins.  Ref: 12612588 Nat Biotechnol, 2003
- METHODS Megakaryocyte progenitor cell subpopulations from cord blood CB and mobilized peripheral blood PBSC were expanded in vitro in the presence of  **mpl**  ligand  [ **THPO** ] .  Ref: 12691920 Exp Hematol, 2003
- Her serum was strongly positive for anti c  **Mpl**   [ **MPL** ]  antibody, and IgG fraction purified from her serum inhibited thrombopoietin  [ **THPO** ]  dependent cell proliferation in vitro.  Ref: 12794833 Arthritis Rheum, 2003
- Furthermore, TPO  [ **THPO** ]  stimulated cellular proliferation appears to be directly correlated with receptor internalization, indicating that internalization of the TPO  [ **THPO** ]   **Mpl**  complex may be essential for normal signal transduction.  Ref: 12623841 Blood< MedlineTA>Blood, 2003
- Recognition of defective platelet thrombopoietin  [ **THPO** ]  receptor  **Mpl**  expression and overexpression of the PRV 1 gene in PV neutrophils has led to studies demonstrating the potential use of these molecular abnormalities as diagnostic markers for PV and for risk stratifying patients with ET for disease conversion to PV or IMF.  Ref: 12682874 Semin Hematol, 2003
- Thus, TPO  [ **THPO** ]  and  **Mpl**  may prove to be effective targets for both diagnosis and therapeutic management of MPD.  Ref: 12682875 Semin Hematol, 2003
- we show a similar modulation by TGF beta1 of a fourth receptor the TPO  [ **THPO** ]  receptor  **MPL** .  Ref: 12725536 Oncol Res, 2003
- These data help identify the receptor motifs involved in TPO  [ **THPO** ]  induced internalization of  **Mpl**  and suggest that  **Mpl**  translocation may be necessary for normal cellular proliferation.  Ref: 12623841 Blood< MedlineTA>Blood, 2003
- Anti c  **Mpl**   [ **MPL** ]  thrombopoietin  [ **THPO** ]  receptor autoantibody induced amegakaryocytic thrombocytopenia in a patient with systemic sclerosis.  Ref: 12794833 Arthritis Rheum, 2003
- When Ba F3 cells were retrovirally transduced with the  **Mpl**  type SAGs, the cells showed Tm and TPO  [ **THPO** ]  dependent growth even without IL 3.  Ref: 12646182 Biochem Biophys Res Commun, 2003
- Because thrombopoietin  [ **THPO** ]  TPO  [ **THPO** ]  and its receptor, c  **Mpl**   [ **MPL** ] , regulate primitive hematopoietic populations, including bone marrow hematopoietic stem cells, we investigated whether TPO  [ **THPO** ]  acts on the hemangioblasts that derive from differentiation of embryonic stem cells in vitro.  Ref: 12743322 Stem Cells, 2003
- Finally, we have demonstrated that upon removal of TPO  [ **THPO** ]  from the supernatant,  **Mpl**  promptly reappears on the cell surface, suggesting that a pool of intracellular  **Mpl**  can be rapidly recycled to the cell surface.  Ref: 12623841 Blood< MedlineTA>Blood, 2003
- In the current study, we examined the in vitro efficacy of modified SAGs containing the thrombopoietin  [ **THPO** ]  TPO  [ **THPO** ]  receptor c  **Mpl**   [ **MPL** ]  gene instead of GCR as a more potent signal generator.  Ref: 12646182 Biochem Biophys Res Commun, 2003
- We aimed to evaluate the proliferative signal transduction events following the activation of c  **mpl**   [ **MPL** ]  and we stimulated B1647 cells with TPO  [ **THPO** ]  40 ng mL for, 15 and 30 minutes.  Ref: 12495897 Haematologica, 2002
- In addition, we constructed various mutant  **Mpl**  type SAGs to abolish the responsiveness to endogenous TPO  [ **THPO** ]  while retaining Tm dependency.  Ref: 12646182 Biochem Biophys Res Commun, 2003
- A corollary of this finding is that TPO  [ **THPO** ]  or the c  **Mpl**   [ **MPL** ]  receptor is a candidate for involvement in the genesis of chronic myeloproliferative diseases MPD, disorders of hematopoietic stem cells.  Ref: 12682875 Semin Hematol, 2003
- We have previously shown that genetically modified marrow cells from mice can proliferate for up to a year in culture in response to CID initiated signals arising from the thrombopoietin  [ **THPO** ]  receptor  **mpl** .  Ref: 12529553 Stem Cells, 2003
- Like CAMT patients, mice lacking the TPO  [ **THPO** ]  receptor c  **Mpl**   [ **MPL** ]  demonstrate a major reduction of early hematopoietic progenitor cells of all lineages.  Ref: 12799278 Ann N Y Acad Sci, 2003
- Suitable consensus sequences were combined in one molecule, resulting in glycosylation analogs of rHuEPO, leptin, and  **Mpl**  ligand  [ **THPO** ] .  Ref: 12612588 Nat Biotechnol, 2003
- The TPO  [ **THPO** ]  receptor c  **MPL**   [ **MPL** ]  abnormalities including reduced  **MPL**  protein levels or defective TPO  [ **THPO** ]  induced signal transduction pathways are the likely mechanisms.  Ref: 12479847 Leuk Res, 2003
- Two alternative hypotheses for the mechanism of regulation have been proposed 1 platelet and or megakaryocyte mass regulate circulating TPO  [ **THPO** ]  levels by binding to TPO  [ **THPO** ]  through TPO  [ **THPO** ]  receptors c  **MPL**   [ **MPL** ] , with subsequent internalization and degradation of the protein.  Ref: 12479847 Leuk Res, 2003
- Here we describe a rare case of SSc accompanied by thrombocytopenia and megakaryocytic hypoplasia, in which autoantibody against thrombopoietin  [ **THPO** ]  receptor c  **Mpl**   [ **MPL** ]  was detected.  Ref: 12794833 Arthritis Rheum, 2003
- In contrast, addition of neutralizing anti c  **Mpl**   [ **MPL** ]  or anti CXCR4 Abs did not influence colony formation, indicating that this synergistic effect was independent of TPO  [ **THPO** ]  or SDF 1.  Ref: 12482498 Exp Hematol, 2002
